# Bilateral inframammary pilonidal sinus: A case report with literature review

**DOI:** 10.1016/j.ijscr.2020.01.003

**Published:** 2020-01-16

**Authors:** Abdulwahid M. Salih, Shvan H. Mohammed, Mohammed Q. Mustafa, Rawand A. Essa, Fahmi H. Kakamad, Tomas M. Mikael, Diyar A. Omar, Karukh K. Mohammed, Hunar Ali Hassan, Masrur S. Aziz, Drood C. Usf, Kayhan A. Najar, Karzan M. Salih

**Affiliations:** a Faculty of Medical Sciences, School of Medicine, University of Sulaimani, Sulaimani, Kurdistan, Iraq; b Kscien Organization, Hamdi Street, Azadi Mall, Sulaimani, Kurdistan, Iraq; c Chara Laboratory, Shahedan Street, Kalar, Kurdistan, Iraq; d Medical Analysis Department, Science Faculty, Tishk University, Erbil, Kurdistan, Iraq; e Erbil Polytechnic University, Shaqlawa Technical Institute, Erbil, Kurdistan, Iraq; f Raparin Laboratory, Nawroz Street, Ranya, Kurdistan, Iraq; g Iraqi Board For Medical Specialties, Sulaimani Center, Sulaymaniyah, Iraq

**Keywords:** Pilonidal, Sinus, Inframammary, Intermammary

## Abstract

**Introduction:**

Pilonidal sinus (PNS) is a chronic inflammatory perianal disorder that rarely occurs outside sacrococcygeal region. The aim of this study is to report an extremely rare case of bilateral inframammary PNS with brief literature review.

**Case report:**

A 25-year-old female presented with a discharging sinuses in both inframammary region for two years. Examination showed multiple discharging sinuses with several centimeters of induration and tenderness. Under general anesthesia, complete excision of the sinuses with primary closure done. Histopathological examinations showed chronic foreign body granuloma surrounding hair shaft pictures consistent with PNS.

**Discussion:**

Inframammary PNS has never been reported in the literature. As with intermammary PNS, in this case also it is associated with obesity and large breasts with tight brassieres. Diagnosis is usually clinical. In contrast to sacrococcygeal PNS, operation under general anesthesia is main treatment modality.

**Conclusion:**

Pilonidal sinus of inframammary region is an extremely rare condition. High index of suspicion is required for diagnosis. Excision with primary closure is the definitive therapy.

## Highlights

• Sacrococcygeal PNS is the commonest variant of the condition.
• However, it can occur in other areas like umbilicus, hand, scalp intermammary, suprapubic, nose, etc.
• Infra mammary PNS is an extremely rare condition, with no previous report.
• This study aims to present and discuss a case of PNS occurring in both infra mammary regions.

## 1 Introduction

PNS is a chronic inflammatory disorder that occurs due to hair involution of epidermis [1]. The most common area is the sacrococcygeal region [2]. It may also occur in rare areas like umbilicus, nose, suprapubic, groin, interdigital web, axilla, clitoris, prepuce and penis [3]. Clinical manifestations are pain, heat and swelling [4]. It is a disease of puberty and adulthood. Male gender is affected more than females by a ratio of 3:1 [5]. Diagnosis of perianal PNS is clinical, however in a case of atypical PNS high index of suspicion is required to suspect the condition [6].

Up to date, 17 cases of intermammary PNS have been reported in the literature, while no inframammary case has been reported [[7], [8], [9], [10], [11]]. This study aims to report a case of bilateral inframammary PNS in line with SCARE guidelines with brief review of intermammary PNS [12].

### 1.1 Patient information

A 25-year-old female presented with multiple discharging sinuses in the both inframammary areas that extends into the intermammary region for two years. Past and family histories were clear. She was non-smoker and single.

### 1.2 Clinical findings

**Fig. 1:**
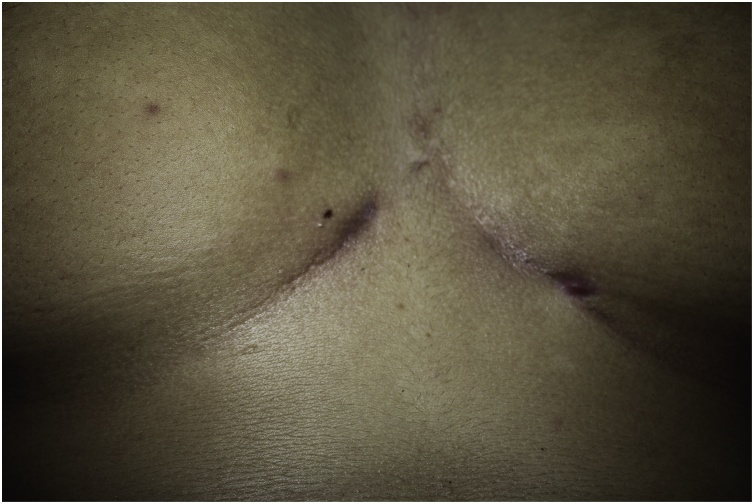
Multiple sinuses involving both inframammary region.

There were multiple discharging sinus extending from left inframammary region, through the intermammary area to the right inframammary sitewith 8 × 9 cm of induration and tenderness (Fig. 1).

### 1.3 Therapeutic intervention

**Fig. 2:**
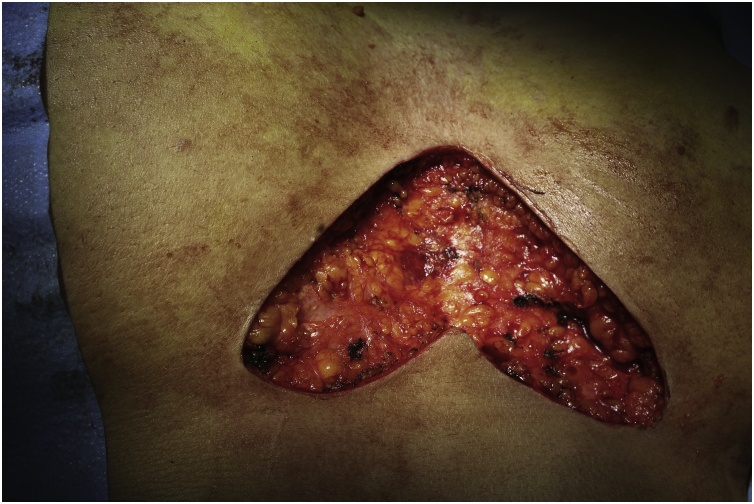
Butterfly incision, resecting all indurated area.

**Fig. 3:**
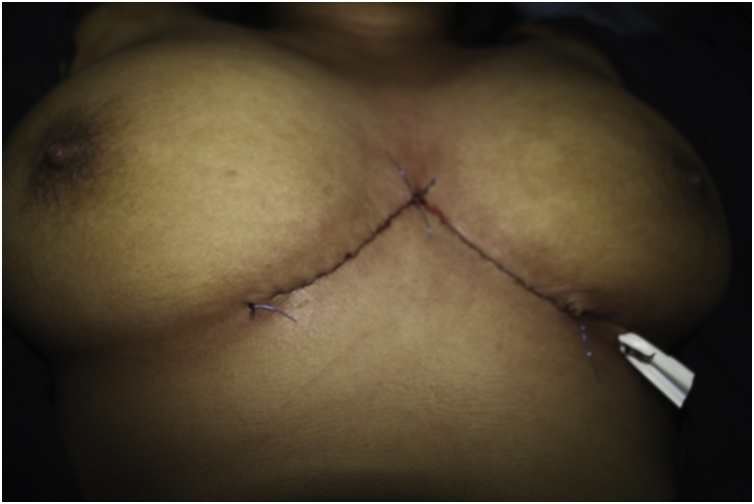
primary closure of the wound.

After preparation for general anesthesia, complete excision of the sinus tracts was performed through a butterfly shaped incision (Fig. 2). Primary closure was done after irrigating the wound with povidone solution and normal saline. Corrugate drain was placed (Fig. 3). Histopathological examinations showed chronic foreign body granuloma surrounding hair shaft pictures consistent with PNS.

### 1.4 Follow-up and outcomes

Two weeks later, the drain was removed. After six months, the scar was healthy, there was no sign of recurrence.

## 2 Discussion

PNS is a suppurative disease that occurs due to hair penetration of epidermis, causing a sequelae of foreign body reaction: inflammation, sinus formation and granulation tissue lined tract [13]. PNS mostly occurs in young people with a male to female ratio of 4:1 [14].

The etiology for PNS was not known until recently (Although the etiology is not well known but nowadays the disease has an acquired etiology) [15]. PNS formation can be attributed to four major reasons: First is penetration of the skin with hair; second, wrinkling of the skin, like in the natal cleft or a scar; third, hormonal and hygienic effects.

The fourth cause is pressure on atypical areas, such as intermammary area by pressure effect of breasts [8,16]. Reports of atypical PNS have increased in the last few decades [16]. A systemic review by Salih et al. found that in more than 300 patients, there are 10 sites for atypical region for PNS to occur other than sacrococcygeal region [16].

In general, the reported risk factors for PNS are hairiness, young age, male gender, prolonged sitting, deep navel and cleft and poor personal hygiene [6]. While the specific risk factors for intermammary PNS is large breast size and tight bra [11]. The current case also reported large breasts and constricting bra.

The differential diagnosis for an atypical PNS is a long list such as hernia, endometriosis, urachal cyst, epidermoid cyst, pyogenic granuloma, dermoid cyst and infected sebaceous cyst [16].

The diagnosis of atypical PNS is not straight forward. Most of intermammary PNS cases can’t be diagnosed preoperatively. Shreef et al. reported 12 cases of intermammary PNS, where only 25% of the cases could be diagnosed preoperatively [11]. In the current case, PNS was suspected preoperatively because of high prevalence of atypical PNS in our locality [1].

There is no standard strategy for management of PNS. It varies from extensive resection to conservative therapy. Injection of a mixture (100 g petroleum jelly (Vaseline) +50 g henna powder (Lawsoniainermis powder) +5 g tetracycline, that was stored at 2 °C–8 °C.) to the PNS site had an extraordinary effect on the patients; they returned to work immediately compared with the operative groups, who stayed at home for at least 10 days [17]. Management of umbilical PNS mostly involves removal of hair and daily dressing without anesthesia, but umbilectomy has been done under general anesthesia [1]. Up to ninety percent of interdigital and hand PNS were treated by surgical excision under general anesthesia [13]. Scalp PNS were treated by excision but one patient required craniotomy [3].

Definite therapeutic approach of intermammary PNS is excision and primary closure [[7], [8], [9], [10], [11]]. The current case was managed by wide local excision with primary repair under general anesthesia.

In conclusion, pilonidal sinus of inframammary area is an extremely rare condition. It is mostly associated with obesity and large breasts with tight brassieres. As intermammary PNS, excision with primary closure is the definitive therapy.

## Sources of funding

No source to be stated.

## Ethical approval

Approval is not necessary for case report in our locality.

## Consent

Consent has been taken from the patient and the family of the patient.

## Author contribution

Abdulwahid M. Salih: Surgeon performed the operation and follow up.

Tomas M. Mikael, Rawand A. Essa, Fahmi H. Kakamad, and Mohammed Q. Mustafa: Writing the manuscript.

Shvan H. Mohammed, Diyar A. Omar, Karukh K. Mohammed, Hunar Ali Hassan, Masrur S. Aziz, Drood C. Usf, Kayhan A. Najar: literature review, final approval of the manuscript.

## Registration of research studies

Not applicable.

## Guarantor

Fahmi Hussein Kakamad.

## Provenance and peer review

Not commissioned, externally peer-reviewed.

## Declaration of Competing Interest

There is no conflict to be declared.
